# Cortical Structure Differences in Relation to Age, Sexual Attractions, and Gender Dysphoria in Adolescents: An Examination of Mean Diffusivity and T1 Relaxation Time

**DOI:** 10.3390/brainsci13060963

**Published:** 2023-06-17

**Authors:** Malvina N. Skorska, Lindsey T. Thurston, Jessica M. Biasin, Gabriel A. Devenyi, Kenneth J. Zucker, M. Mallar Chakravarty, Meng-Chuan Lai, Doug P. VanderLaan

**Affiliations:** 1 Child & Youth Psychiatry, Centre for Addiction and Mental Health, Toronto, ON M6J 1H4, Canada; malvina.skorska@utoronto.ca (M.N.S.);; 2 Department of Psychology, University of Toronto Mississauga, Mississauga, ON L5L 1C6, Canada; 3 Cerebral Imaging Centre, Douglas Mental Health University Institute, Montreal, QC H4H 1R3, Canadamallar.chakravarty@douglas.mcgill.ca (M.M.C.); 4 Department of Psychiatry, McGill University, Montreal, QC H3A 1A1, Canada; 5 Department of Psychiatry, Temerty Faculty of Medicine, University of Toronto, Toronto, ON M5T 1R8, Canada; 6 Department of Biological and Biomedical Engineering, McGill University, Montreal, QC H3A 2B4, Canada; 7 The Margaret and Wallace McCain Centre for Child, Youth & Family Mental Health, Azrieli Adult Neurodevelopmental Centre, Campbell Family Mental Health Research Institute, Centre for Addiction and Mental Health, Toronto, ON M6J 1H4, Canada; 8 Department of Psychiatry and Autism Research Unit, The Hospital for Sick Children, Toronto, ON M5G 1X8, Canada; 9 Autism Research Centre, Department of Psychiatry, University of Cambridge, Cambridge CB2 8AH, UK; 10 Department of Psychiatry, National Taiwan University Hospital and College of Medicine, Taipei 100229, Taiwan

**Keywords:** gender dysphoria, mean diffusivity, T1 relaxometry, sexual orientation, adolescence, brain development, brain tissue microstructure, partial least squares, structural MRI

## Abstract

Recent research found that the combination of masculine gender identity and gynephilia was associated with cortical T1 relaxation time, which is considered to reflect gray matter density. We hypothesized that mean diffusivity (MD), a diffusion tensor imaging metric that reflects the degree to which water movement is free versus constrained, in combination with T1 relaxation time would provide further insight regarding cortical tissue characteristics. MD and T1 relaxation time were measured in 76 cortical regions in 15 adolescents assigned female at birth who experience gender dysphoria (GD AFAB) and were not receiving hormone therapy, 17 cisgender girls, and 14 cisgender boys (ages 12–17 years). Sexual orientation was represented by the degree of androphilia–gynephilia and the strength of sexual attraction. In multivariate analyses, cortical T1 relaxation time showed a weak but statistically significant positive association with MD across the cortex, suggesting that macromolecule-rich cortical tissue also tends to show water movement that is somewhat more constrained. In further multivariate analyses, in several left frontal, parietal, and temporal regions, the combination of shorter T1 relaxation time and faster MD was associated with older age and greater gynephilia in GD AFAB individuals and cisgender boys and with stronger attractions in cisgender boys only. Thus, for these cortical regions in these groups, older age, gynephilia, and stronger attractions (cisgender boys only) were associated with macromolecule-rich tissue in which water movement was freer—a pattern that some prior research suggests is associated with greater cell density and size. Overall, this study indicates that investigating T1 relaxation time and MD together can further inform how cortical gray matter tissue characteristics relate to age and psychosexuality.

## 1. Introduction

A growing domain in the neuroimaging literature has examined brain features related to variations in human sexual orientation and gender identity [1,2]. Aspects of cortical structure—most notably, cortical volume, thickness, and surface area—have often been the focus of these studies [3,4,5,6,7]. A recent multi-modal neuroimaging study of 12- to 17-year-olds by Skorska et al. [8] examined cortical thickness, surface area, and T1 relaxation time—the latter measure being a previously unconsidered metric that provides information pertaining to the relative presence vs. absence of macromolecules or the free-water content within brain tissue [9]. Using a multivariate approach that included these three metrics, there was a widespread pattern across cortical regions that was mostly driven by T1 relaxation time. Specifically, among cisgender (i.e., sex assigned at birth and gender identity align) boys, older age, stronger sexual attractions, and greater gynephilic attractions (i.e., sexual attractions towards females) were associated with shorter T1 relaxation times. Similarly, in adolescents assigned female at birth who met criteria for gender dysphoria (GD AFAB; i.e., distress related to an incongruence between sex assigned at birth and experienced gender), older age and greater gynephilic attractions were associated with shorter T1 relaxation times. In contrast, these patterns were not evident among cisgender adolescent girls. Further, there was no main effect of gender identity on T1 relaxation time, but rather the associations with T1 depended on the combination of gender identification, sexual orientation, and age.

Based on these findings and given that a shorter T1 relaxation time is purported to reflect macromolecule-rich tissue or tissue that has less free-water content [9], Skorska et al. [8] inferred that denser cortical gray matter might be a brain characteristic that develops during adolescence among masculine-identifying gynephilic individuals regardless of sex assigned at birth. If this inference was accurate, it would help to further clarify the brain features related to variations in human sexual orientation and gender identity.

To follow up on and extend the findings of Skorska et al. [8], it may be useful to consider other measures that could, in addition to T1 relaxation time, provide information relevant to gray matter density. For example, given that T1 relaxation time is a less commonly acquired measure, future investigations of cortical tissue density and variations in sexual orientation and gender would be aided if there was some reasonable alternative brain metric that correlated with T1 relaxation time and could inform tissue density. Structural brain metrics that can be examined in existing data sets of sexually and/or gender-diverse participants would be particularly useful as they would offer a more immediate avenue for further examining variations in neural tissue density. Diffusion-weighted imaging (DWI), which has been more commonly acquired in the neuroimaging literature on sexual orientation and gender identity to investigate white matter and to examine sex or gender differences [6,10,11,12,13,14,15,16], might provide a viable alternative.

In particular, mean diffusivity (MD) is one metric that can be derived from DWI [17,18] and applied to data sets that include sexually diverse and/or gender-diverse participants. As MD reflects the degree to which water moves freely vs. is constrained by surrounding tissue [18,19], we hypothesize that MD can serve as an alternate measure of tissue density that should correlate positively with T1 relaxation time. For example, a shorter T1 relaxation time might reflect the presence of many macromolecules in the tissue, increasing the density of the tissue [9]. A shorter T1 relaxation time may also reflect less free-water content, indicating that water molecules are less free to diffuse (i.e., are more restricted) [20]. In such a case, water may move less freely between cells, which could also be reflected by a slower MD. Thus, if the hypothesis holds and T1 relaxation time and MD correlate as predicted, then either metric could be employed to provide information that helps to further understand the cortical tissue density differences reported by Skorska et al. [8].

Alternatively, T1 relaxation time and MD could correlate negatively, which would lead to other inferences about cortical gray matter tissue. A prior study that combined T1 relaxometry and DWI to investigate temporal cortical gray matter found that both shorter T1 relaxation times and less directionality of water movement, as indicated by lower fractional anisotropy (FA) values, were associated with greater neuronal cell size and density [21]. MD is a metric that describes the rate of water diffusion, whereas FA is a fraction wherein the shape of the tensor ellipsoid is used to infer the directionality of the water movement [22,23,24,25]. Thus, “fast” and “slow” are used to describe MD, whereas “high” and “low” are used to describe FA. FA and MD are often inversely related as both low FA and fast MD indicate less directional, freer water movement [18]; however, FA appears to have less reproducibility in gray matter, whereas the reproducibility of MD is considered to be relatively better [26], suggesting that it may be preferable to consider MD’s relationship with T1 relaxation time. As such, if a shorter T1 relaxation time were negatively associated with MD (i.e., a faster MD indicating less-restricted water movement), it might suggest the presence of greater cell size and density in cortical gray matter (as per the pattern reported by [21]).

In an adolescent sample that mostly overlapped with the sample examined by Skorska et al. [8], the present study examined cortical gray matter MD and T1 relaxation time. The relation of these metrics to one another as well as their associations with age, sexual attractions, and gender identity were investigated. We reasoned that one possibility was that a shorter T1, reflecting macromolecule-rich tissue, would be expected to correspond with slower MD, reflecting water diffusion that is more constrained by surrounding tissue (i.e., T1 relaxation time and MD should be positively correlated). Further, if this was the case, then MD and T1 relaxation time might relate to age, sexual attractions, and gender in a similar fashion. An alternative possibility was that MD and T1 relaxation time would show an inverse relationship with one another, as well as with respect to the manner in which they relate to age, sexual attractions, and gender identity. In either case, the resulting patterns would help form inferences about the properties of cortical gray matter tissue that could then direct future research.

## 2. Materials and Methods

### 2.1. Participants

The present study focused on 46 adolescents who participated from 2014 to 2018. The sample included 15 AFAB adolescents who met *Diagnostic and Statistical Manual of Mental Disorders, Fifth Edition* (DSM-5) [27] criteria for GD and were not receiving puberty blockers or gender-affirming hormone therapy, 17 cisgender adolescent girls, and 15 cisgender adolescent boys. Data from five additional participants were removed: data from one GD AFAB adolescent were removed due to excessive artifacts from a metal orthodontic expander, data from one GD AFAB adolescent were removed due to an unusable MD map, data from one cisgender boy were removed due to excessive head motion during T1-weighted (T1w) scans, and data from one GD AFAB adolescent and one cisgender boy were removed because the images needed to calculate the T1 maps were unusable. Forty-seven of the participants were included in Skorska et al. [8], and forty-nine participants were included in Skorska et al. [28].

The participants were 12 to 17 years old, with a mean age of 15.30 years (SD = 1.66). Over half of the individuals in the sample were of “European”/“White” ethnicity (*n* = 26, 56.5%) and the remainder were other ethnicities (*n* = 20, 43.5%). An inclusion criterion for all participants was an age of 12 to 17 years. The GD AFAB participants must have been assessed by a clinician and must have met criteria for GD. Exclusion criteria were any form of hormone therapy (not including oral contraceptives), having a known disorder of hormone regulation or sex development, any contraindications to MRI (e.g., pregnancy), oral orthodontics, any head trauma, or a lack of English-language proficiency for the completion of the study measures. For the cisgender individuals, additional exclusion criteria were any previous mental health diagnosis, participation in a special education class in school, involvement with a child protection agency, and feeling uncomfortable with their sex assigned at birth or identifying or wishing to identify as a member of another gender.

The GD AFAB participants were recruited from the Gender Identity Service (GIS) at the Centre for Addiction and Mental Health (CAMH) or from a clinician specializing in GD in a private practice. At the GIS, all prospective participants within the specified age range were informed of the study. If GD adolescents were interested in participating, a member of the study team not involved in their clinical care shared information about the study, answered questions, performed a screening, and obtained informed assent and/or consent to participate. Cisgender participants were recruited from the community via advertisements posted online (Kijiji, www.kijiji.ca; Facebook, www.facebook.com) and on bulletin boards in the Greater Toronto Area that described the study as being about brain development and gender. Word of mouth was also used to distribute information about the study to cisgender participants.

### 2.2. Procedure

Eligibility screenings were conducted in person or over the phone. If eligible, verbal assent was provided by adolescents aged between 12 and 15 years old, and a parent/guardian provided informed consent. Informed consent was provided by adolescents who were 16 or 17 years old. The study parts consisted of a blood draw of about 20 mL, a 1 h MRI session, and the completion of a brief intelligence assessment and questionnaire package. Some GD participants’ data were obtained from clinical assessments. Most participants completed all study procedures on the same day, and the order of study parts varied. After participation, the adolescents were thanked, received an honorarium of CAD 20 per hour, and received compensation for their travel expenses. Only measures relevant to the aims of this study are reported. The study was conducted in accordance with the Declaration of Helsinki, and the protocol was approved by the Research Ethics Board of CAMH (#145-2013).

### 2.3. Measures

All numerical values for the variables reported and most of the code used are available from Borealis [29]. Where applicable, the means were computed for those who responded to at least 75% of the items.

#### 2.3.1. Age

Age in months (rounded to the nearest month) was calculated as the participant’s date of birth minus the date on which the consent form was signed.

#### 2.3.2. Gender Dysphoria

The Gender Identity/Gender Dysphoria Questionnaire for Adolescents and Adults (GIDYQ-AA) [30,31] assesses self-reported gender identity and GD with good discriminant validity and clinical utility [30,31,32,33]. The male version and parallel female version were completed by cisgender boys, cisgender girls, and GD AFAB participants, respectively. Each of the 27 items were rated on a 5-point Likert-type scale ranging from 1 (always) to 5 (never) regarding the past 12 months. An example of a male version item is: “In the past 12 months, have you felt unhappy being a boy?” The threshold for a likelihood of meeting GD criteria is a GIDYQ-AA score < 3.00 [33]. A mean score was calculated, with a lower score indicating greater GD.

#### 2.3.3. Sexual Orientation

The Erotic Response and Orientation Scale (EROS) assesses self-reported sexual attractions and sexual fantasies over the past 6 months with good discriminant validity [32,34,35]. Eight items address attractions/fantasies toward boys/men (i.e., androphilia, e.g., “How often have you noticed you had any sexual feelings [even the slightest] while looking at a boy/man?”), and eight items utilize similar language to assess attractions/fantasies toward girls/women (i.e., gynephilia). Each item was rated on a 5-point Likert-type scale ranging from 1 (not at all) to 5 (almost every day). Mean androphilia and mean gynephilia scores were derived for each participant such that higher scores reflected more attractions/fantasies. The internal consistency was high (Cronbach’s alpha: androphilia = 0.95, gynephilia = 0.95).

In previous studies, mean gynephilia scores were subtracted from mean androphilia scores or were used as separate variables [32,33]; however, we recognized that doing so would ignore an important component of sexual attractions captured by EROS scores: the strength of the attractions (i.e., no attraction to many attractions, regardless of the target or degree of attractions). Furthermore, the mean androphilia and mean gynephilia scores were not significantly correlated with each other (see Section 3.1). Thus, we operationalized sexual orientation by deriving two metrics to characterize the two aspects of sexual orientation that can be deduced from EROS scores: the strength of attractions and the degree of androphilia–gynephilia (see also [8]). The mean androphilia and gynephilia scores (range of 1 to 5) were used to create a vector, $\vec{AndroGyne}$, by calculating the magnitude or length of the vector to represent strength of attractions and the phase, θ, of the vector to represent the valence of sexual attractions along the androphilia–gynephilia dimension. The following formula was used to calculate the magnitude of the vector (i.e., the strength of sexual attractions) such that a longer vector represents stronger attractions:

(1) $$\sqrt{{\left((androphiliascore\right)}^{2}+{\left(gynephiliascore\right)}^{2})}$$

Phase (i.e., the degree of androphilia–gynephilia) was calculated using the following formula:

(2) $$\left(arccos\left(\frac{androphiliascore}{magnitude}\right)\right)*\left(\frac{180{}^{\circ}}{\pi }\right)$$

**Figure 1:**
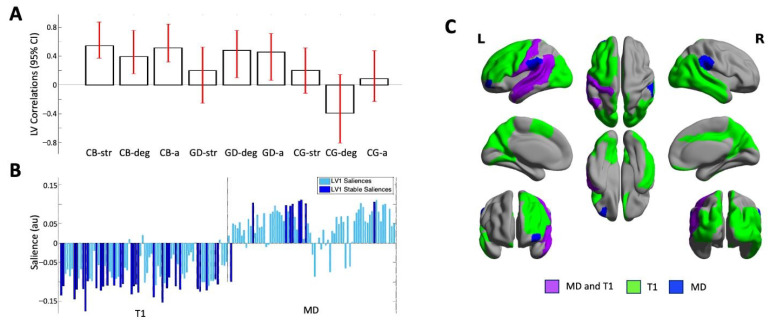
Results of the one significant latent variable from the PLS analysis with both T1 and MD values. (Panel **A**) Correlations of brain scores with behavior (± 95% confidence interval, CI). This latent variable identified similar and stable correlations in cisgender boys (CBs) and GD AFAB individuals (GDs) which were stable for age (CB-a; GD-a) and degree of androphilia-gynephilia (CB-deg; GD-deg) in both groups and for strength of sexual attractions in the CBs (CB-str). No correlations were stable in the cisgender girls (CG-str, CG-deg, CG-a). Scatterplots of brain scores by behavioral measures for all participants can be found in Figure S4 (Supplementary Material). (Panel **B**) Brain saliences for all ROIs for T1 and MD. The majority of brain saliences were negative, indicating that in cisgender boys and GD AFAB individuals, shorter T1s and slower MDs (one region) correlated with older age and stronger gynephilia, and in cisgender boys only, stronger attractions. For the remainder of the stable MD regions, a faster MD value correlated with age and sexual orientation in cisgender boys and GD AFAB individuals. (Panel **C**) Surface projection of stable ROIs: green = T1 relaxation time only, blue = MD only, purple = MD and T1; created with BrainNet Viewer (version 1.7, Beijing; https://www.nitrc.org/projects/bnv/) [53]. L = left; R = right.

Phase is symmetrically distributed ± 34° around 45° such that an 11° phase represents exclusive androphilia, a 79° phase represents exclusive gynephilia, and a 45° phase represents equal scores on androphilia and gynephilia (e.g., asexuality or ambiphilia). See Figures 1 and 2 in Ref. [8] for a visual depiction of this operationalization of EROS scores and the data distribution, which aligned with the majority of participants in the current study. The Supplementary Materials contains descriptive statistics (Table S1) and frequency distributions (Figure S2) of the mean androphilia and mean gynephilia variables by group. Frequency distributions of the strength of attractions and the degree of androphilia–gynephilia variables by group are in Figure S3. In addition, scatterplots of brain scores by the strength of attractions and degree of androphilia–gynephilia variables for all participants are shown in Figure S4.

### 2.4. MRI Methods

#### 2.4.1. Image Acquisition

The participants were scanned at CAMH in Toronto, ON, Canada, on a GE MR750 3T magnetic resonance scanner (General Electric, Milwaukee, WI, USA) with an 8-channel head coil (General Electric, 8HR BRAIN, GE Standard 8-Channel Head Coil). The T1-weighted (T1w) images were acquired with a T1 BRAVO pulse sequence in the sagittal plane: inversion time = 650 ms, echo time = 3 ms, repetition time = 6.8 ms, flip angle = 8°, field of view = 23 cm, 256 mm × 256 mm matrix, 200 isotropic 0.9 mm thick slices, and acquisition time = 4:41 min. The calculation of the T1 maps with B1 corrections required four acquisitions using sagittal spoiled-gradient echo (SPGR) pulse sequences. These scans included two fast-SPGR scans (repetition time = 10.6 ms, echo time = ~ 4.4 ms, and acquisition time = 2:59 min each) with 1 mm isotropic resolution (field of view = 25.6 cm, 160 slices) at two flip angles (14° and 3°). These two images will be referred to as “Flip14” and “Flip3,” respectively, and were used for T1 mapping using the variable flip angle (VFA) method [36,37]. To account for B1 inhomogeneities, B1 maps were computed using an extrapolation to signal null as per the method of slopes (MoS) [38] from two SPGR scans (repetition time = 50–60 ms, echo time = 5 ms, and acquisition time = 2:24 min each) with low, 4 mm isotropic resolutions (field of view = 25.6 cm; 40 slices) and two flip angles (130° and 150°). The total acquisition time for the four scans was 9:46 min. Diffusion-weighted images were acquired using a 2D spin echo planar imaging (EPI) sequence with axial slices in the anterior–posterior direction: 2 mm isotropic resolution, b = 1000 mm^2^/s, 60 diffusion-weighted, 5 non-diffusion-weighted images, and total acquisition time = 9:41 min. The T2-weighted (T2w) images were acquired using an FSE-XL pulse sequence in the axial plane: flip angle = 125°, field of view = 22 cm, 256 mm × 256 mm matrix, 3 mm thick slices, and total acquisition time = 1:46 min.

#### 2.4.2. Image Processing

The T1w images were pre-processed using the minc-bpipe-library (https://github.com/CobraLab/minc-bpipe-library.git, accessed on 1 May 2023) on SciNet [39,40]. Briefly, the images were realigned to roughly align with the Montreal Neurological Institute (MNI) template, signal intensity non-uniformity was corrected [41], and the brain was extracted [42]. Quality checking of the images for intensity non-uniformity, motion, and the quality of brain extraction was performed and the extracted brains were manually edited to remove obvious brain extraction errors.

All four SPGR images used to create the T1 maps were reoriented into the space defined as halfway between the Flip3 and Flip14 images using FLIRT (FMRIB’s Linear Image Registration Tool) from the FSL (FMRIB Software Library, version 5.0.9) tool library (https://fsl.fmrib.ox.ac.uk/fsl/fslwiki/). Specifically, the “halfway flirt” command from FSL’s SIENA pipeline was used. The Flip3 image was brain-extracted using FSL’s BET (Brain Extraction Tool), and the resulting mask was used to extract the brain of the Flip14 image. The T1 maps were then calculated and calibrated as described in Chavez [43]. In this procedure, B1 maps were generated using the MoS [38], and the T1 maps were computed using the VFA method with a B1 correction incorporated to obtain the true flip angle voxel-wise [37].

In Skorska et al. [8], the Anatomical Automatic Labelling (AAL) atlas [44] was used to delineate 76 regions of interest (ROIs) (38 per hemisphere) across the cortical gray matter. To extract the ROI data from the T1 maps, the reoriented Flip14 image was registered to the brain-extracted T1w image from the minc-bpipe-library pipeline. The resulting transformation matrix was used to move the T1 map into T1w space using FSL’s FLIRT. Brain-extracted T1w images from the minc-bpipe-library were registered to the 1 mm MNI template using antsRegistration (version 2.1.0) [45,46]. The resultant matrix was then inverted and used to move the AAL template from MNI space to T1w space using ANTs’ antsApplyTransforms with the generic label option. Each registration step and transformed image was manually inspected for registration errors.

The T1 data were extracted only from gray matter voxels in each ROI. FSL’s FAST (FMRIB’s Automated Segmentation Tool) was used to segment the brain-extracted T1w images from the minc-bpipe-library pipeline into gray matter, white matter, and cerebral spinal fluid, allowing for partial volume estimates and disabling the bias field correction. Using an in-house MATLAB (2017b, version 9.3.0.713579) script, T1 data were extracted for the 76 AAL atlas ROIs. The T1 data were included only for voxels in which the gray matter probability was >0.40 (i.e., the voxel contained at least 40% gray matter). A histogram of T1 values was plotted for each ROI using T1s 500 ms < T1 < 5000 ms and a bin width of 30 ms (see Figure S1 in Skorska et al. [8]). The distributions were generally unimodal (some ROIs included white matter, which led to a small peak at lower T1) but were often positively skewed (partly due to partial volume effects between the gray matter and cerebral spinal fluid). To avoid overestimating T1 in the ROI, the T1 mode, rather than the mean, was used to represent the T1 value in the ROI. This was accomplished by fitting a spline function to the histogram and finding the T1 value at the peak. Histograms of the T1 values were spot checked for anomalies.

Diffusion-weighted images were first corrected for susceptibility distortion due to the EPI acquisition. The mean non-diffusion weighted (b = 0) image was non-linearly registered to a non-EPI based T2-weighted (T2w) image using FSL’s FNIRT (FMRIB’s Nonlinear Image Registration Tool). The warpres parameter constrained the transformation to the y-plane only to account for posterior–anterior distortion of the EPI images along the phase-encoding direction. Next, the images were corrected for eddy current distortions using FSL’s eddy algorithm [47]. As the diffusion weighted images were in T2w space while the T1 maps and AAL atlas were in T1w space, the MD files were moved into T1w space. First, the T2w image was moved into T1w space using FSL’s FLIRT (FMRIB’s Linear Image Registration Tool) and applyxfm, utilizing the mutualinfo cost non-default setting. Utilizing FSL’s BET (Brain Extraction Tool) with a nondefault setting of an f of 0.2 and the mask option, the brain and a brain mask were extracted from the raw T2w image. The distortion-corrected diffusion-weighted data (in T2w space) were then processed using FSL’s DTIFIT to fit each voxel with a diffusion tensor model. The brain mask from the BET output was utilized here. Maps were calculated for the FA, MD, axial diffusivity, and radial diffusivity, but only the MD maps were used in the current study. Last, using the matrix from the T2w to T1w transformation, the MD files from DTIFIT were moved from T2w space to T1w space using FSL’s FLIRT with the applyxfm option.

To extract the MD values for each ROI, a MATLAB (R2021a, version 9.10.0.1602886) script similar to the script for extracting T1 values was created. Thus, the MD data were extracted for the 76 AAL atlas ROIs and included only voxels in which the gray matter probability was > 0.40. A histogram of MD values was plotted for each ROI using MD values between 0.0005 and 0.004 with a bin width of 50 (see Figure S1 in the Supplementary Materials). The distributions were generally unimodal, and some had a slight positive skew, indicating that the mean MD value could serve as a good representation of MD in the ROI. Histograms of the MD values were spot checked for anomalies.

### 2.5. Statistical Analyses

#### 2.5.1. Group Differences in Demographic and Psychosexual Variables

Using SPSS version 28 (Armonk, NY), group differences for age, the GIDYQ-AA, and EROS variables were examined with a one-way analysis of variance (ANOVA). In the presence of a significant omnibus effect, post hoc comparisons were conducted with least significant difference (LSD) tests. The GIDYQ-AA had a significant Levene’s test, so the Games–Howell post hoc test, robust to heterogeneity of variance, was used for this measure. A two-tailed critical *p*-value of 0.05 was used.

#### 2.5.2. Multivariate Correlation of Regional MD and T1 Relaxation Time

To investigate the association between MD and T1 in a multivariate analysis, we ran a nested model in Mplus version 8.8 [48]. MD was the dependent variable, T1 was the independent variable, and participant ID was the cluster variable, provided that each participant had MD and T1 values. A fixed slope and random intercept default model were specified, which allowed us to test the association between T1 and MD values across cortical regions and participants [49]. Standardized results are reported.

#### 2.5.3. Multivariate Associations of Regional MD and T1 Relaxation Time with Age, Sexual Orientation, and Gender

Partial least squares (PLS), a multivariate statistical technique, [50,51] was used to examine the commonalities and differences in brain–behavior correlations across the three groups. PLS is an appropriate analytic approach when the number of observations exceeds the number of samples or participants, as was the case here. The behavior variables were the two EROS variables (i.e., strength of attractions and degree of androphilia–gynephilia) and age, and the analysis assessed the degree to which they correlated with the brain measures. PLS uses a singular value decomposition of a data matrix to produce latent variables (LVs) with three components: the correlations of brain scores with behavior measures, which reflect the group contrast (*v*), the brain saliences/weights (*u*), and the measures of the strength of those relationships (*s*). The data matrix was created as a single row for each participant containing 76 T1 values and 76 MD values. To minimize the impact of any differences in data distributions as well as differences in metric-specific ranges of values on the results, all brain data were normalized by converting them to *z*-scores within each ROI (i.e., across all participants) [52].

Permutation tests (1000 samples) provide an exact probability and assess the number of times the strength of the permuted latent variables exceeds the observed strength. Bootstrap resampling (1000 samples) estimate the standard error of each brain salience, assessing the reliability of their contributions to the observed pattern. The ratio of the salience to its standard error approximates a *z*-score, and for all analyses, a threshold of ± 3 was used to identify stable, reliable contributions. Confidence intervals (CI, 95%) around the point estimates are calculated using the bootstrap sampling distribution. “Brain scores” are the projection (dot-product) of the brain saliences (*u*) with a participant’s data and provide an indication of how strongly each participant reflects the contrast identified on the LV. The PLS analysis was conducted using the PLS software available at https://www.rotman-baycrest.on.ca/index.php?section=84 (v. 6.1311050, Toronto, ON), using MATLAB (R2021a, version 9.10.0.1602886, Natick, MA).

## 3. Results

### 3.1. Differences in Demographic and Psychosexual Variables

Descriptive statistics are shown by group in Table 1. The skewness and kurtosis values, which were less than |2|, demonstrated no extreme deviations from normality.

Table 1 also shows the omnibus results from the ANOVAs for age, the GIDYQ-AA, the strength of attractions, and the degree of androphilia–gynephilia. The results did not differ from Skorska et al. [8] or Skorska et al. [28] and are thus summarized here. Details can be found in the Supplementary Materials. For age, the main effect of group was not significant (Table 1). As expected, there was a significant effect for the GIDYQ-AA such that the GD AFAB group scored significantly lower than both the cisgender girls and cisgender boys, who did not differ from each other. The threshold for the likelihood of meeting GD criteria is a GIDYQ-AA score < 3.00 [33]. The lowest score on the GIDYQ-AA from the cisgender participants was 4.48, indicating that none of the cisgender participants met this threshold; the highest score in the GD AFAB group was 3.04, with all other GD AFAB participants scoring below the threshold.

Regarding sexual orientation, a main effect of group was found for degree of androphilia–gynephilia, whereas strength of attractions showed no significant group differences (Table 1). For degree of androphilia–gynephilia, cisgender boys were more gynephilic than both GD AFAB individuals and cisgender girls. GD AFAB individuals were more gynephilic than cisgender girls. Cisgender boys tended to be gynephilic, cisgender girls tended to be androphilic, and GD AFAB individuals had a range of sexual attractions, with a cluster of individuals in the ambiphilic or asexual ranges.

Androphilia scores were not related to gynephilia scores across participants or within each group. The strength of attractions was also not related to the degree of androphilia–gynephilia across participants or within each group. The strength of attractions was significantly correlated with age across all participants, within cisgender boys, and within cisgender girls but not within GD AFAB individuals. The degree of androphilia–gynephilia was not significantly correlated with age across all participants or within any group.

### 3.2. Multivariate Correlation of Regional MD and T1 Relaxation Time

A nested regression model was run in Mplus. T1 significantly predicted MD (estimate = 0.089, SE = 0.034, and *p* = 0.008). Given the direction of the association was positive, a shorter T1 was related to slower MD.

### 3.3. Multivariate Associations of Regional MD and T1 Relaxation Time with Age and Sexual Attractions by Group

The PLS analysis identified one significant LV (*p* = 0.000) that accounted for 62.75% of the cross-block covariance between the ROI data and the two EROS variables and age across the groups. Cisgender boys and GD AFAB individuals shared similar brain–behavior correlations, with no stable correlations in the cisgender girls. For the cisgender boys, the strength of attractions, degree of androphilia–gynephilia, and age were stably related to the ROI data; for the GD AFAB group, similar correlations were found for degree of androphilia–gynephilia and age (Figure 1, Panel A).

Most of the T1 brain saliences were negative and most of the MD brain saliences were positive, indicating that the T1 metrics were inversely related to the behavioral measures and the MD metrics were positively related to the behavioral measures. In six regions distributed across the left frontal, temporal, and parietal lobes (Table 2; Figure 1, Panel C), in GD AFAB individuals and cisgender boys, shorter regional T1 relaxation times and slower MD correlated with older age, stronger gynephilia, and—for cisgender boys only—stronger attractions. In the left orbital part of the middle frontal gyrus, faster MD correlated with older age and stronger gynephilia in cisgender boys and GD AFAB individuals, and stronger attractions in cisgender boys but no T1 association were observed in this region (Table 2; Figure 1, Panel C). Across all measures, the brain–behavior correlations in cisgender boys and GD AFAB individuals were most stably expressed in 38 regions, 29 of which reflected regional correlations with T1 and 9 of which reflected regional correlations with MD (Figure 1, Panel B). The stable regions were distributed throughout the lobes of the brain, particularly for T1 (Table 2; Figure 1, Panel C).

## 4. Discussion

The present study investigated the relation of cortical gray matter T1 relaxation time and MD to each other as well as their associations with age, sexual attractions, and gender identity in an adolescent sample. In doing so, we assessed whether MD might be a reasonable proxy of cortical gray matter density, which appeared to be greater among masculine-identifying gynephilic individuals in later adolescence when Skorska et al. [8] examined cortical T1 relaxation time. If MD were a reasonable metric for this purpose, then it could perhaps be informative in other samples to further assess the associations between cortical gray matter characteristics and age, sexual attractions, and gender identity. An alternative possibility was that MD complements T1 relaxation time to provide more detailed insight regarding cortical gray matter tissue characteristics and their relations to age, sexual attractions, and gender.

Our analyses yielded some findings that suggest investigating cortical gray matter MD is informative. Across cortical regions, there was a statistically significant positive correlation between T1 relaxation time and MD. The positive correlation between T1 relaxation time and MD suggests that the latter is also related to the relative presence of macromolecules and thus tissue density; however, this correlation had a weak effect size, suggesting that T1 and MD only provide somewhat overlapping information. In addition, there was limited evidence that slower MD was associated with older age, stronger gynephilia, and stronger sexual attractions among masculine-identifying individuals (i.e., this pattern was only seen in one cortical region; see Figure 1). As such, the present findings suggest MD alone is unlikely to show much association between slower cortical MD and these age- and psychosexuality-related characteristics.

Instead, MD appeared to best serve as a complementary measure of T1 relaxation time in the context of investigating cortical structure in relation to age, sexual attractions, and gender identity. These brain structure metrics were (primarily) inversely associated with age and sexual attractions among cisgender boys and GD AFAB individuals. More specifically, our results showed that in six cortical regions across the left frontal, parietal, and temporal lobes, the combination of shorter T1 relaxation time and faster MD tended to be more likely among masculine-identifying individuals who reported stronger gynephilic attractions and were in later adolescence regardless of sex assigned at birth. This pattern likely reflects macromolecule-rich tissue with relatively freer movement of water, which can inform more detailed inferences about the cortical tissue characteristics associated with this age-related psychosexual phenotype.

To our knowledge, only one prior study in humans examined cortical gray matter using T1 relaxometry, DWI, and a histological analysis of cellular characteristics [21]. In cortical tissue, it was found that both a shorter T1 relaxation time and a lower FA value, which is often inversely associated with MD (i.e., associated with faster MD) [18], were associated with greater neuronal cell size and density. Thus, using the findings of Goubran et al. [21] to guide interpretation, the pattern of shorter T1 relaxation time and faster MD in the present study could reflect greater neuronal size and density. To further evaluate this possibility, it will be important to test whether this complementary pattern between T1 relaxation time and MD replicates in other samples. In addition, there are other DWI approaches that could be employed. Some prior research has used DWI-derived metrics to make inferences about cellular density via restriction spectrum imaging by separating the diffusion-weighted signal into components representing restricted and hindered diffusion and using the combined information to infer the degree of cellular density [54,55]. Another example is neurite orientation dispersion and density imaging (NODDI), which can use multi-shell DWI data to model microstructural features of tissue such as neuronal density [56]. Such information could also be used to evaluate the types of age- and psychosexuality-related differences in cortical gray matter studied here.

Thus, the current findings help point to some directions for future research. If available, T1 relaxation time can be used to follow up on the original findings reported by Skorska et al. [8]. Such studies will clarify whether greater gray matter density is indeed associated with the combination of masculine gender identity and gynephilia regardless of sex assigned at birth. If researchers are interested in exploring whether there are alternative metrics that inform gray matter tissue density and/or cellular characteristics, then it could be beneficial to further evaluate the association of T1 relaxation time with MD and to examine certain other DWI-derived metrics (for examples of such metrics, see [54,55,56]).

Future research could also address some of the present study’s limitations. We had relatively small sample sizes. In addition, representation across sexual attractions and gender identity could be improved. The cisgender boys and girls examined here were disproportionately gynephilic and androphilic, respectively, and the study lacked a group of individuals assigned male at birth who experienced gender dysphoria. The lack of such participants limits the conclusions that can be drawn. For example, given the lack of cisgender gynephilic girls, it is unclear whether gynephilia is associated with a cortical pattern of slower T1 and faster MD regardless of whether one’s gender expression is masculine or feminine. The study also lacked a comparison of transgender youth who identified with a binary gender (i.e., girl or boy) to those with a nonbinary gender identity. In addition, our sample was limited to adolescents. Given that our findings suggest cortical gray matter density in masculine gynephilic individuals covaries with increased age over adolescence, this pattern of psychosexual-related neural characteristics might be more evident in adult samples.

It will also be important for future research to investigate the factors that influence variations in cortical structure. Cortical volume, thickness, and surface area appear to vary with biological factors (e.g., androgens) thought to influence cortical development [57,58,59,60], and such biological factors have also been implicated in the development of sexual orientation and gender identity [2,59,61,62]. In contrast, the development of cortical gray matter tissue density and its relevance to age- and psychosexual-related differences is less well understood. For example, gray matter density has been reported to be higher in adolescent girls than boys, both at the whole-brain level and across several regions of the brain [63], although in another study, sex/gender were not associated with T1 relaxation time in gray matter [64]. Our previous study [8], which relied on a sample largely overlapping with that of the present study, also did not find a sex assigned at birth difference in T1 relaxation time. Regarding these mixed findings across studies, it is unclear whether the inconsistencies are due to not including and accounting for sexual orientation and gender expression diversity in the study design in [63], differences in sample size between the studies in question, or the use of different brain metrics to examine gray matter density (macromolecule presence inferred from T1 relaxation time in [8,64] vs. gray matter estimates based on T1-weighted images in [63]). Regarding factors affecting MD, MD in gray matter has been shown to be heritable, with some significant genetic correlations between cortical MD and cortical thickness in regions across the brain shown in one study [19]. The development of gray matter is influenced in part by myelination [65], and myelination is also affected by estrogens and androgens [57,66,67,68,69,70]. However, the exact mechanisms and how they relate to sexual attractions and gender have not been fully elucidated.

In sum, greater cortical gray matter density might be a brain characteristic of masculine gynephilic individuals that emerges over adolescent development regardless of sex assigned at birth. T1 relaxation time can be used to further investigate this association. Investigating T1 relaxation time in combination with MD and/or other DWI-derived metrics [54,55,56] may further inform gray matter tissue/cellular characteristics associated with age and psychosexuality. Important directions for future research include the further evaluation of the relationships between T1 relaxation time and DWI-derived metrics. Studies could also benefit from employing larger samples with better representation of individuals across the sexual orientation and gender identity spectrums, as well as samples that include adults. Lastly, better understanding the factors that influence cortical gray matter density could provide insight into psychosexuality-related brain development.

## Figures and Tables

**Table 1:** Descriptive statistics for demographic and psychosexual variables.

	Cisgender Boys	GD AFAB	Cisgender Girls	*F* (*df*)	*p*
*n*	14	15	17		
Age (months) ^a^					
M	184.93	193.07	191.71	0.68 (2, 43)	0.510
SD	25.61	14.65	19.00		
Range	147–216	162–216	152–214		
GIDYQ-AA					
M	4.91	2.17	4.90	778.90 (2, 43) ^d^	<0.001
SD	0.12	0.33	0.15		
Range (1–5) ^b^	4.63–5.00	1.74–3.04	4.48–5.00		
Strength of attractions					
M	3.25	2.98	2.83	0.53 (2, 43) ^e^	0.591
SD	1.28	1.34	0.76		
Range (1.41–7.07) ^c^	1.70–5.37	1.41–6.05	1.41–4.44		
Degree of androphilia–gynephilia					
M	64.60	46.99	30.94	25.67 (2, 43)	<0.001
SD	9.29	15.56	13.18		
Range (11–79) ^b^	43.96–75.55	17.35–75.55	14.04–68.20		

Note. GIDYQ-AA = Gender Identity/Gender Dysphoria Questionnaire for Adolescents and Adults. ^a^ For age in years, cisgender boys (M = 15.41, SD = 2.13, range = 12.25–18); GD AFAB (M = 16.09, SD = 1.22, range = 13.5–18); and cisgender girls (M = 15.98, SD = 1.58, range = 12.67–17.83). Note that the 18-year-olds had not yet reached their 18th birthday. ^b^ Absolute range. ^c^ Possible range for magnitude and phase (θ) based on EROS scores with absolute range of 1 to 5. ^d^ A significant Levene’s test (*p* = 0.043) warrants reporting of the robust tests of equality of means via the Welch test statistic (2, 26.42) = 463.98, *p* < 0.001, and the Brown–Forsythe test statistic (2, 23.70) = 769.19, *p* < 0.001. ^e^ A significant Levene’s test (*p* = 0.022) warrants reporting of the robust tests of equality of means via the Welch test statistic (2, 24.79) = 0.59, *p* = 0.564, and the Brown–Forsythe test statistic (2, 34.57) = 0.51, *p* = 0.606.

**Table 2:** Stable ROIs contributing to PLS analysis.

Hem.	ROI		
	Frontal Lobe	T1	MD
L	Inferior Frontal Gyrus: Opercular Part	x	-
L	Inferior Frontal Gyrus: Triangular Part	x	-
L	Middle Frontal Gyrus	x	-
L	Middle Frontal Gyrus: Orbital Part	-	x
L	Precentral Gyrus	x	-
L	Rolandic Operculum	x	x
L	Superior Frontal Gyrus: Dorsolateral	x	-
L	Supplementary Motor Area	x	-
	Parietal Lobe		
L	Angular Gyrus	x	x
R	Angular Gyrus	x	-
L	Postcentral Gyrus	x	x
L	Precuneus	x	-
R	Precuneus	x	-
L	Supramarginal Gyrus	-	x
R	Supramarginal Gyrus	-	x
	Temporal Lobe		
L	Heschl Gyrus	x	x
R	Heschl Gyrus	x	-
R	Inferior Temporal Gyrus	x	-
L	Middle Temporal Gyrus	x	x
R	Middle Temporal Gyrus	x	-
L	Superior Temporal Gyrus	x	x
R	Superior Temporal Gyrus	x	-
	Occipital Lobe		
L	Cuneus	x	-
R	Cuneus	x	-
L	Inferior Occipital Gyrus	x	-
R	Inferior Occipital Gyrus	x	-
L	Lingual Gyrus	x	-
L	Middle Occipital Gyrus	x	-
R	Middle Occipital Gyrus	x	-
L	Superior Occipital Gyrus	x	-
R	Superior Occipital Gyrus	x	-
	Insula and Cingulate Gyri		
R	Median Cingulate and Paracingulate Gyri	x	-

Note. PLS = partial least squares; T1 = T1 relaxation time; MD = mean diffusivity; Hem. = hemisphere; ROI = region of interest; L = left; R = right.

## Data Availability

All numerical values for variables reported and most of the code used are available from Borealis at https://borealisdata.ca/dataset.xhtml?persistentId=doi:10.5683/SP3/QVPE0F, accessed on 1 May 2023 (see also [29]).

## Supplementary Materials

The following are available online at: https://www.mdpi.com/article/10.3390/brainsci13060963/s1, Table S1: Descriptive statistics for the raw EROS variables by group, Figure S1: Examples of MD histograms; Figure S2: Histograms of raw EROS scores by group; Figure S3: Histograms of EROS scores transformed into strength of attractions and androphilia–gynephilia variables by group; Figure S4: Scatterplots showing the brain score-behavior correlations for all groups and behavioral measures from the T1 and MD partial least squares analysis.
