# Supplementary material for: Cortical Structure Differences in Relation to Age, Sexual Attractions, and Gender Dysphoria in Adolescents: An Examination of Mean Diffusivity and T1 Relaxation Time
Source: Brain Sci. 2023 Jun 17;13(6):963. doi: 10.3390/brainsci13060963 (PMC10296103; doi:10.3390/brainsci13060963)
Supplement: Supplementary file 1 [file brainsci-13-00963-s001.zip › brainsci-2411615-supplementary.pdf]

**SUPPLEMENTARY MATERIALS**

**Title**: Cortical Structure Differences in Relation to Age, Sexual Attractions, and Gender Dysphoria in Adolescents: An Examination of Mean Diffusivity and T1 Relaxation Time

**Authors**: Skorska, M. N., Thurston, L. T., Biasin, J. M., Devenyi, G. A., Zucker, K. J., Chakravarty, M. M., Lai, M.-C., & VanderLaan, D. P.

**Mean Diffusivity (MD) Histograms**

Examples of MD histograms for four regions of interest (ROIs) in four participants are shown in Figure S1. These are representative of most of the histograms across participants and ROIs. The mean value of the histogram was utilized as representative of the MD value for each ROI for each participant.

**Differences in Demographic and Psychosexual Variables**

Omnibus results are presented in the main text and in Table 1. Here we provide details for mean differences (MDiff) and correlations. For the GIDYQ-AA the GD AFAB group scored significantly lower than both the cisgender girls (MDiff = -2.73, *SE* = 0.09, $p < 0.001$) and cisgender boys (MDiff = -2.74, *SE* = 0.09, $p < 0.001$), who did not differ from each other (MDiff = 0.01, *SE* = 0.05, $p = 0.970$). For degree of androphilia-gynephilia, cisgender boys were more gynephilic than both GD AFAB (MDiff = 17.61, *SE* = 4.84, $p < 0.001$) and cisgender girls (MDiff = 33.66, *SE* = 4.70, $p < 0.001$). GD AFAB were more gynephilic than cisgender girls (MDiff = 16.05, *SE* = 4.61, $p = 0.001$).

Androphilia scores were not related to gynephilia scores across participants, ($r = -0.09$, $p = 0.576$, $n = 46$), within cisgender boys ($r = 0.43$, $p = 0.129$, $n = 14$), within GD AFAB ($r = 0.25$,

*p* = 0.370, *n* = 15), or within cisgender girls (*r* = -0.16, *p* = 0.553, *n* = 17). Strength of attractions was not related to degree of androphilia-gynephilia across participants (*r* = 0.05, *p* = 0.753, *n* = 46), within cisgender boys (*r* = 0.24, *p* = 0.418, *n* = 14), within GD AFAB participants (*r* = -0.15, *p* = 0.604, *n* = 15), or within cisgender girls (*r* = -0.35, *p* = 0.163, *n* = 17). Strength of attractions was significantly correlated with age across all participants (*r* = 0.53, $p < 0.001$, *n* = 46), within cisgender boys (*r* = 0.87, $p < 0.001$, *n* = 14), within cisgender girls (*r* = 0.58, *p* = 0.014, *n* = 17), but not within GD AFAB (*r* = 0.20, *p* = 0.485, *n* = 15). Degree of androphilia-gynephilia was not significantly correlated with age across all participants (*r* = 0.02, *p* = 0.918, *n* = 46), within cisgender boys (*r* = 0.39, *p* = 0.168, *n* = 14), within GD AFAB (*r* = 0.26, *p* = 0.352, *n* = 15), and within cisgender girls (*r* = -0.01, *p* = 0.958, *n* = 17).

**Raw EROS Scores**

Descriptive statistics for mean androphilia and mean gynephilia by group are provided in Table S1. Supplementary Figure S2 contains frequency distributions of the raw EROS scores by group and Supplementary Figure S3 contains frequency distributions of the EROS scores transformed into the degree of androphilia-gynephilia and strength of attractions variables, by group.

Table S1. Descriptive statistics for the raw EROS variables by group.

| | Cisgender Boys | GD AFAB | Cisgender Girls |
|---|---|---|---|
| *n* | 14 | 15 | 17 |
| Mean Androphilia | | | |
| *M* | 1.33 | 1.98 | 2.40 |
| *SD* | 0.74 | 1.24 | 0.86 |
| Range (1-5)[a] | 1.00 - 3.63 | 1.00 - 4.75 | 1.00 - 4.13 |
| Mean Gynephilia | | | |
| *M* | 2.91 | 2.05 | 1.36 |
| *SD* | 1.18 | 1.03 | 0.49 |
| Range (1-5)[a] | 1.38 - 4.88 | 1.00 - 3.88 | 1.00 - 2.50 |

*Note*. [a]Absolute range.

## Supplementary Figures

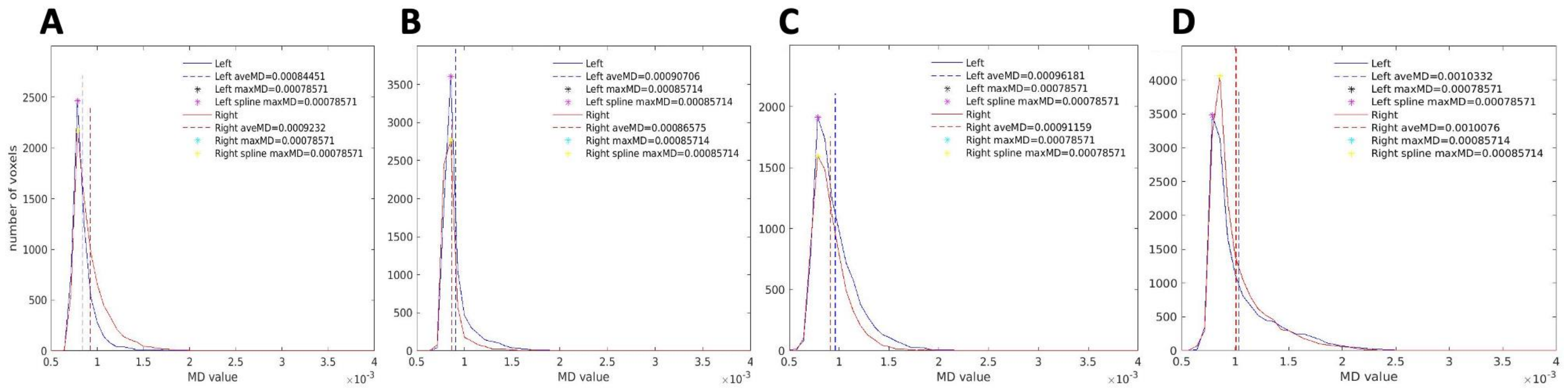


**Supplemental Figure S1. Examples of MD Histograms.** MD histograms from the left and right hemispheres of the angular gyrus (Panel A), anterior cingulate and paracingulate gyri (Panel B), cuneus (Panel C), and superior frontal gyrus (Panel D) are shown. The distribution of MD values was generally slightly positively skewed for the majority of regions across participants.

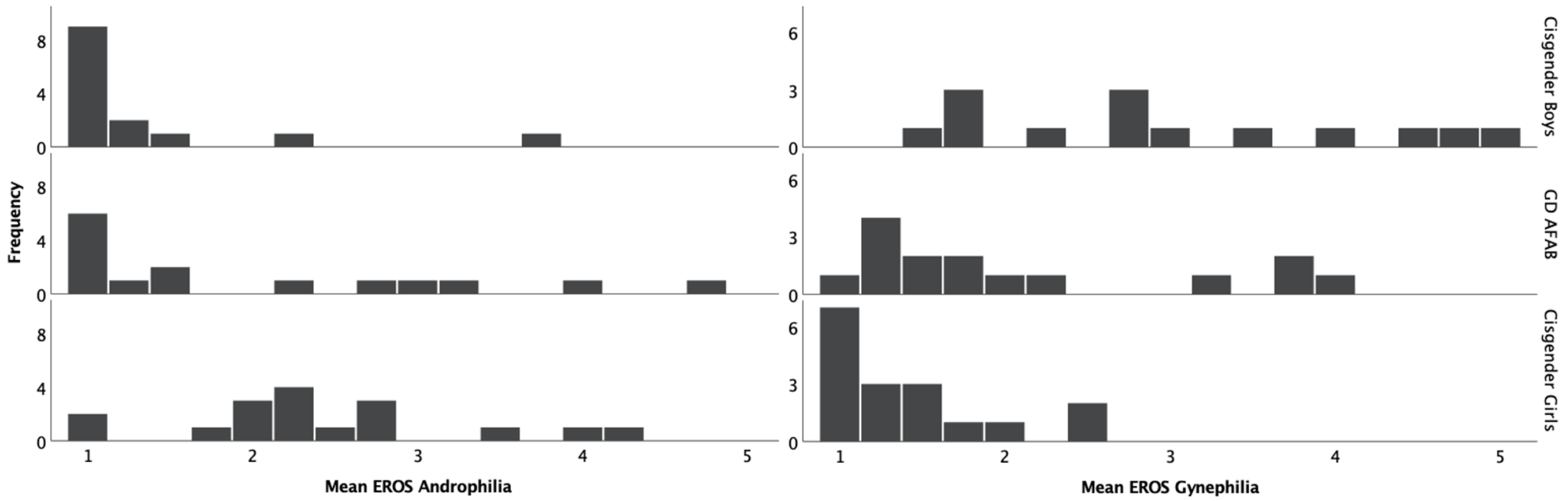


**Supplemental Figure S2. Histograms of Raw EROS Scores, by Group.** EROS = Erotic Response and Orientation Scale. Androphilia represents attractions/fantasies towards men or boys and gynephilia represents attractions/fantasies towards women or girls. Higher scores represent greater attraction or fantasies.

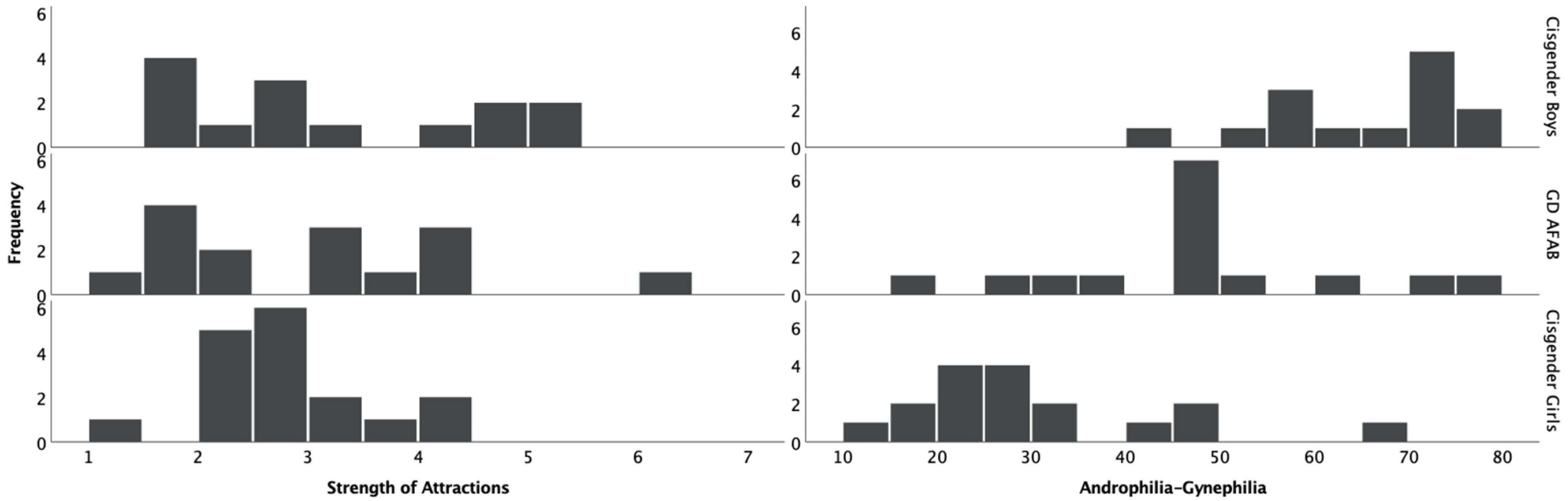


**Supplemental Figure S3. Histograms of EROS Scores Transformed into Strength of Attractions and Degree of Androphilia-Gynephilia Variables, by Group.** EROS = Erotic Response and Orientation Scale. Androphilia represents attractions/fantasies towards men or boys and gynephilia represents attractions/fantasies towards women or girls. The strength of attractions and degree of androphilia-gynephilia variables were calculated from EROS androphilia and gynephilia scores. For strength of attractions, higher numbers indicate stronger attractions, independent of the target of attractions. For degree of androphilia-gynephilia, 11 represents exclusive androphilia, 79 represents exclusive gynephilia and 45 represents no or equal (i.e., ambiphilic) attractions.

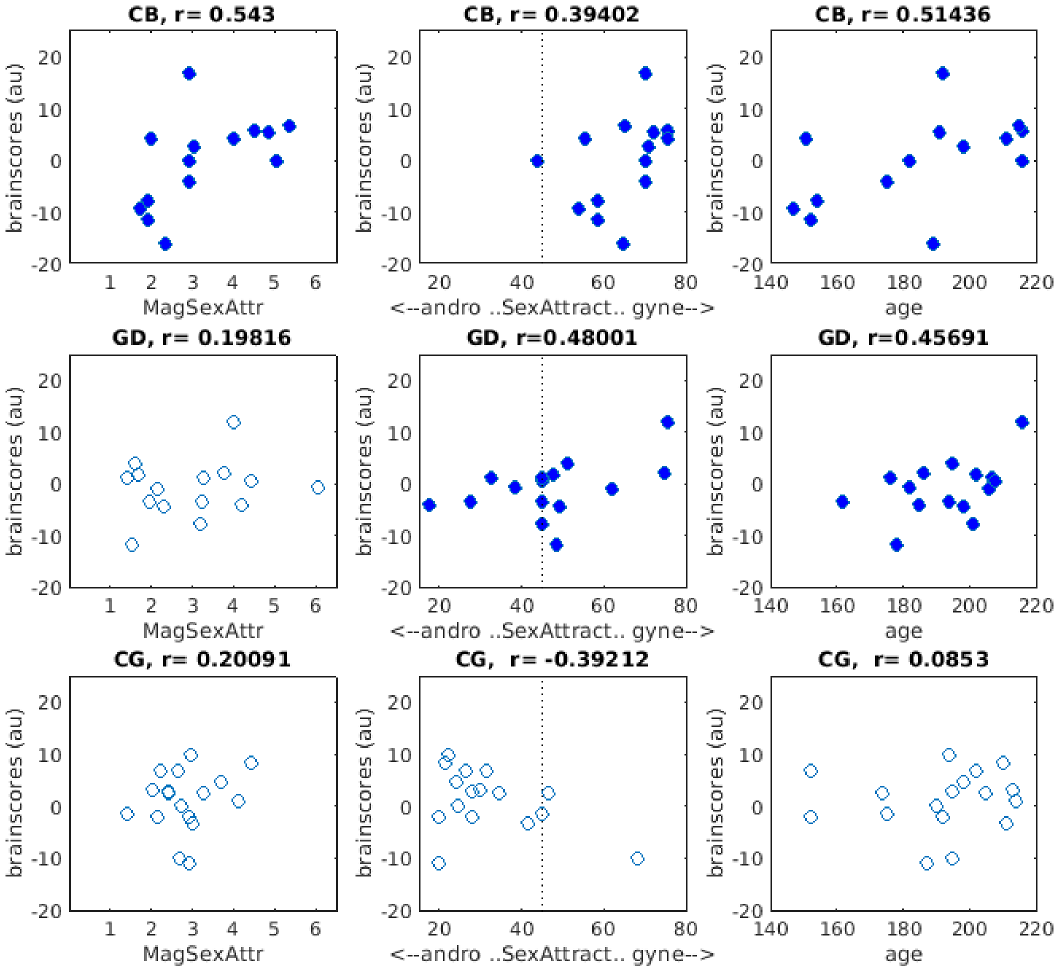


**Supplemental Figure S4. Scatterplots showing the brain score-behavior correlations for all groups and behavioral measures from the T1 and MD partial least squares analysis.** These correlations are also shown in Fig. 1, Panel A as bar plots with 95% CIs. Left column is the strength of sexual attractions (MagSexAttr). Center column is the direction of sexual attraction (SexAttr) with a range from exclusively androphilic (11°) to exclusively gynephilic (79°). The dotted line at 45°indicates ambiphilic or asexual and the range is indicated by the dot-dashed line. Right column is age (months). The strength of the brain-behavior correlation is indicated at the top of each plot, and stable correlations are indicated by filled markers. CB = cisgender boys, GD = GD AFAB, CG = cisgender girls.
